# Correction: Brucellosis testing patterns at health facilities in Arusha region, northern Tanzania

**DOI:** 10.1371/journal.pone.0344498

**Published:** 2026-03-06

**Authors:** AbdulHamid Settenda Lukambagire, Gabriel Mkulima Shirima, Damas Davis Shayo, Coletha Mathew, Richard B. Yapi, Christopher Jacob Kasanga, Blandina Theophile Mmbaga, Rudovick Reuben Kazwala, Jo E. B. Halliday

The sixth author’s name is incorrect. The correct name is: Christopher Jacob Kasanga.

There are errors in the Funding statement. The correct Funding statement is as follows: ASL, GMS, CM, RBY, BTM, RRK were supported by the DELTAS Africa Initiative Afrique One- ASPIRE scholarship scheme (Afrique One-ASPIRE/DEL-15–008, http://afriqueoneaspire.org). This work was also supported by the Biotechnology & Biological Sciences Research Council, the Department for International Development, the Economic & Social Research Council, the Medical Research Council, the Natural Environment Research Council and the Defence Science & Technology Laboratory, under the Zoonoses and Emerging Livestock Systems (ZELS) program grant number BB/L018845 (http://www.bbsrc.ukri.org/). The funders had no role in study design, data collection and analysis, decision to publish, or preparation of the manuscript.
