# Supplementary material for: Brucellosis testing patterns at health facilities in Arusha region, northern Tanzania
Source: PLoS One. 2022 Mar 23;17(3):e0265612. doi: 10.1371/journal.pone.0265612 (PMC8942238; doi:10.1371/journal.pone.0265612)
Supplement: S1 Fig — Data completeness is calculated assuming that all facilities operated for the full study period and accounts for the fact that only five months of data were included for 2018. (DOCX) [file pone.0265612.s001.docx]

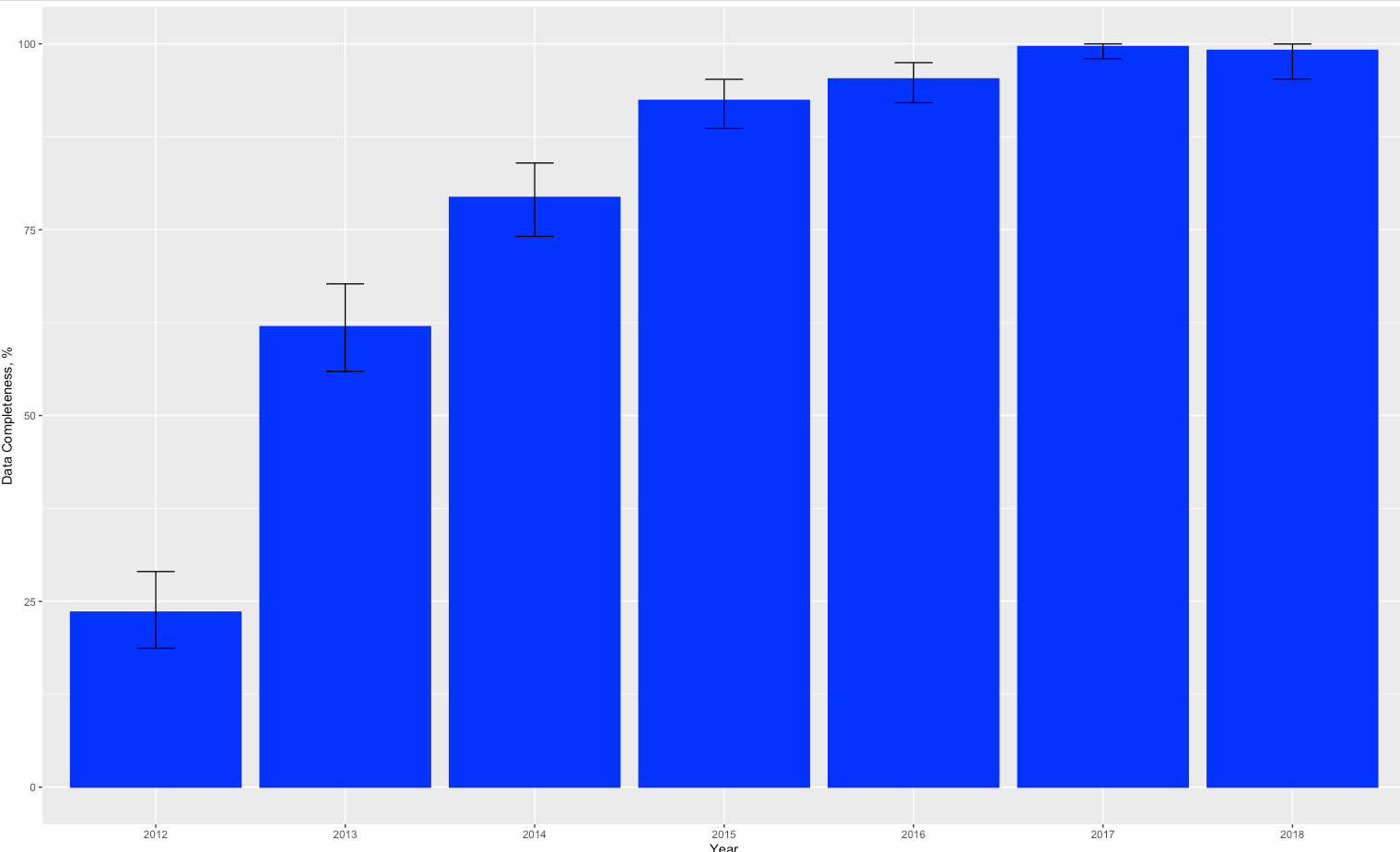


**S1 Fig. Graph showing data completeness for the 23 facilities contributing data over the seven years of the data collection period.** Data completeness is calculated assuming that all facilities operated for the full study period and accounts for the fact that only five months of data were included for 2018.
