# Supplementary material for: Brucellosis testing patterns at health facilities in Arusha region, northern Tanzania
Source: PLoS One. 2022 Mar 23;17(3):e0265612. doi: 10.1371/journal.pone.0265612 (PMC8942238; doi:10.1371/journal.pone.0265612)
Supplement: S1 Table — CI- Confidence interval. (DOCX) [file pone.0265612.s002.docx]

**S1 Table: Facility characteristics for the study period from January 2011 to May 2018 inclusive (n = 24 facilities); CI- Confidence interval**

| Variable | Category | n/N | Percentage (95% CI) |
| --- | --- | --- | --- |
| Ownership | Faith-based organization | 8/24 | 33.3 (15.6 – 55.3) |
|  | Government | 13/24 | 54.2 (32.8 – 74.4) |
|  | Private | 3/24 | 12.5 (2.7 – 32.4) |
| Clinical diagnosis done | Yes | 5/24 | 20.8 (7.1 – 42.2) |
|  | No | 19/24 | 79.2 (57.8 – 92.9) |
| Brucellosis testing done | Yes | 21/24 | 87.5 (67.6 – 97.3) |
|  | No | 3/24 | 12.5 (7.1 42.2) |
| Reason for not testing | No reagent | 14/21 | 66.7 (43.0 – 85.4) |
|  | No reagent controls | 4/21 | 19.0 (5.4 – 42.0) |
|  | Don’t report brucellosis | 3/3 | 100 (0.3 – 100) |
| Brucellosis reagent currently used for testing | Eurocell | 17/21 | 81.0 (58.1 – 96.0) |
|  | Fortress | 9/21 | 42.9 (21.8 – 66.0) |
|  | Genuine Biosystem | 7/21 | 33.3 (14.6 – 57.0) |
|  | Arkray | 4/21 | 19.0 (5.4 – 42.0) |
|  | Rose Bengal Test | 0/21 | - |
| Brucellosis reagent reported previous use | Eurocell | 10/21 | 47.6 (25.7 – 70.2) |
|  | Fortress | 8/21 | 38.1 (18.1 – 61.6) |
|  | Genuine Biosystem | 8/21 | 38.1 (18.1 – 61.6) |
|  | Arkray | 4/21 | 19.0 (5.4 – 42.0) |
|  | Rose Bengal Test | 1/21 | 4.8 (0.1 – 23.8) |
| Reagent supplier | Supplier 1 (Arusha) | 17/21 | 81.0 (58.1 – 96.0) |
|  | Supplier 2 (Arusha) | 7/21 | 33.3 (14.6 – 57.0) |
| Reagent purchase cost in TZS | 30,000 | 12/21 | 57.1 (34.0 – 78.2) |
|  | 35,000 | 1/21 | 4.8 (0.1 – 23.8) |
|  | 45,000 | 10/21 | 47.6 (25.7 – 70.2) |
|  | 50,000 | 8/21 | 38.1 (18.1 – 61.6) |
|  | 60,000 | 4/21 | 19.0 (5.4 – 42.0) |
|  | 65,000 | 3/21 | 14.3 (3.0 – 36.3) |
| Test cost to patient in TZS (Cat) | 2000 – 3000 | 12/21 | 57.1 (34.0 – 78.2) |
|  | 3000 – 5000 | 11/21 | 52.4 (29.8 – 74.3) |
|  | 5000 – 10000 | 1/21 | 4.8 (0.1 – 23.8) |
| Do you se controls in test runs | Yes | 5/21 | 23.8 (8.2 – 47.2) |
|  | No | 16/21 | 76.2 (52.8 – 91.8) |
| Control material used | Kit provided | 3/5 | 60.0 (14.7 – 94.7) |
|  | Positive serum | 2/5 | 40.0 (5.3 – 85.3) |
|  | Both | 2/5 | 40.0 (5.3 – 85.3) |
| Performed QA for each run | Yes | 5/21 | 23.8 (8.2 – 47.2) |
|  | No | 16/21 | 76.2 (52.8 – 91.8) |
| Run serial dilutions | Yes | 1/21 | 4.8 (0.1 – 23.8) |
|  | No | 20/21 | 95.2 (76.2 – 99.9) |
| Test result reported | Pos or Neg | 15/21 | 71.4 (47.8 – 88.7) |
|  | Pos (*B. abortus, B. melitensis*) or Neg | 5/21 | 23.8 (8.2 – 47.2) |
|  | Titres | 1/21 | 4.8 (0.1 – 23.8) |
| Any test re-run | Yes | 8/21 | 38.1 (18.1 – 61.6) |
|  | No | 13/21 | 61.9 (38.4 – 81.9) |
| Reason for rerun test | Result verification | 5/8 | 62.5 (24.5 – 91.5) |
|  | Confirm recovery | 3/8 | 37.5 (8.5 – 75.5) |
